# Frequency-dependent impairment calibration and estimation for a 96 GBaud coherent optical transceiver

**DOI:** 10.1038/s44172-023-00147-3

**Published:** 2024-01-05

**Authors:** Longquan Dai, Ziheng Zhang, Zicai Cao, Shuchang Yao, Tianming Li, Yudi Fu, Jing Dai, Yaqin Wang, Ming Luo, Xi Xiao, Mengfan Cheng, Qi Yang, Ming Tang, Deming Liu, Lei Deng

**Affiliations:** 1 Wuhan National Laboratory for Optoelectronics and School of Optical and Electronic Information, Huazhong University of Science and Technology, Wuhan, 430074 China; 2 Fiberhome Telecommunication Technologies Co., LTD, Wuhan, 430073 China; 3 China Information and Communication Technologies Group Corporation, 430074 Wuhan, China; 4 National Information Optoelectronics Innovation Center, 430074 Wuhan, China; 5 Shenzhen Huazhong University of Science and Technology Research Institute, Shenzhen, 518000 China

**Keywords:** Fibre optics and optical communications, Optoelectronic devices and components

## Abstract

For 800 Gbps/λ and beyond optical transmission systems, frequency-dependent impairments (FDI) degrade coherent optical transceiver (CO-TRx) performance severely. Calibration and compensation of such FDI in factories includes amplitude/phase frequency response (AFR/PFR), skew, and ripple for both transmitters(Tx) and receivers (Rx). However, due to the polarization rotation and phase rotation effects in optical link, the separation and extraction of FDI from different polarization or I/Q tributaries is challenging. Here we report a FDI calibration method based on orthogonal separation scheme and frequency domain analysis. The proposal can simultaneously characterize and separate the Tx/Rx sides FDI including AFR, PFR, and time skew of four tributaries. Finally, the effectiveness is demonstrated by transmitting a 96 GBuad Nyquist-16QAM signal on a 64 GBaud-class CO-TRx.

## Abstract (web summary)

Frequency dependent impairment degrades the transmission performance but its accurate calibration is still challenging due to the complex polarization. Dai, Zhang and colleagues report a calibration method able to simultaneously calibrate various frequency-dependent impairments in coherent optical transceivers, with a measured bit error rate improvement from 1.61e^-1 to 4.41e^-2 after calibration.

## Introduction

With the increasing demand for optical network capacity, coherent optical transceiver (CO-TRx) with a single carrier rate exceeding 800 Gbps is being extensively and deeply researched. Transmission of signals with a baud rate higher than 100 GBaud and a modulation format higher than 64QAM is the most intuitive way to increase the system capacity^1–5^.

However, when increasing the baud rate and modulation format, the frequency-dependent impairment (FDI) becomes a prominent factor affecting the transmission performance. The FDI can be summarized as transmitter (Tx) and receiver (Rx) side amplitude-frequency response (AFR), phase frequency response (PFR), and time skew between I/Q tributaries (IQ skew) and X/Y polarization state (XY skew). When the payload signals transmit in a high-speed system where the impact of AFR/PFR and IQ/XY skew play a major role, the performance of the conventional digital signal processing (DSP) algorithms will be significantly degraded^6,7^. From the perspective of the impairment mechanism, FDI will exhibit different gain, ripple, and phase characteristics in the variance of frequencies within the margin of device bandwidth, which will cause inter-symbol interference. And the benefit of precise frequency response compensation for CO-TRx has been widely demonstrated^8,9^. For the time skew between different transmitted tributaries, the same skew value will have a more serious impact on CO-TRx with higher baud rates and higher modulation formats. It has been reported that the IQ skew tolerance for 16QAM and 64QAM signals with 1 dB SNR penalty under given conditions are less than 11% and 4.2% of the symbol period, corresponding to 1.1 ps and 0.42 ps for 100 GBaud transmission^7^. In addition, the existence of XY skew can greatly affect the polarization demodulation process and degrade the transmission performance of high-speed CO-TRx^10^. Since the AFR/PFR and IQ/XY skew of the coherent optical transceiver are static or very slow drifting parameters, the calibration of FDI becomes a suitable option.

According to the functions, FDI calibration methods can be divided into the Tx-only, the Rx-only, and both Tx/Rx-sides, as shown in Table 1. It can be observed that most of the calibration studies focus on one-side FDI. The combined optical-electrical effect makes it impossible to obtain the AFR/PFR of CO-TRx by using a vector network analyzer alone. Therefore, a high-resolution optical spectrum analyzer (HR OSA) is applied to obtain the AFR at the Tx or Rx sides^11,12^. However, HR OSA will increase the cost and cannot solve the problem of time skew. To reduce the cost and consider the skew issue, calibration methods using only the photodiode (PD) built-in modulator are proposed, and the Tx AFR/PFR or IQ skew can be derived from the beating of the transmitted signal^13–16^. The reconfigurable interference and equalizer-based machine learning are also used to obtain the Tx skew values^17,18^. In most cases, the Rx IQ/XY skew can be derived from the tap distribution of the converged Rx equalizers^19,20^.

**Table 1:** Summary of frequency-dependent impairments calibration methods.

Reference	Transmitter side				Receiver side				Required additional resources	Year
	IQ skew	XY skew	AFR	PFR	IQ skew	XY skew	AFR	PFR		
^17^	√									2019
^11^			√						HR OSA	2013
^13^			√							2016
^16^				√					PD (< 1 GHz)	2021
^18^	√	√								2016
^14^	√		√							2022
^15^	√		√							2017
^12^							√		HR OSA	2019
^19^					√					2017
^20^					√					2013
^21^	√				√					2019
^22^	√				√					2022
^23^	√				√					2022
^24^	√				√					2023
^25^		√				√				2019
^26^	√		√		√		√		PD (< 1 GHz)	2022
**This work**	√	√	√	√	√	√	√	√	PD (< 1 GHz)	2023

High-accuracy and multi-functional FDI calibration of CO-TRx on both Tx/Rx sides is rarely achieved due to the high difficulty. The FDI from the Tx side or Rx side does not exist independently, they usually occur together. Complex time-domain noise such as azimuth rotation, laser phase noise, frequency offset effect, and rotation of the state of polarization (RSOP) can combine with crosstalk between adjacent ports, resulting in deep mixing and overlapping of impairments. The presence of frequency offset will alter the overall frequency response of the CO-TRx. Azimuth rotation and laser phase noise will distribute the signals from the same Tx port to different Rx ports, causing crosstalk between I/Q tributaries. The RSOP effect will mix the signals of different polarization states, together with the azimuth rotation effect, the signal from one Tx port may be sent to any Rx port. Moreover, the crosstalk in the circuit will directly distribute the high-frequency parts of the signals between adjacent ports. All of these factors will inevitably increase the difficulty in separating and calibrating the mixed FDI on both the Tx and Rx sides.

Despite the difficulties, several studies in recent years have succeeded in separating some FDI from the Tx and Rx sides. In^21^, researchers utilize the effect of laser frequency offset and phase noise to gradually compensate the Rx and Tx, thereby separating the IQ skew on different sides of the transceiver. Then, the interleaved multi-tone signals proposed in^22^ can separate the impairments from different Tx and Rx tributaries in the frequency domain, so that Tx and Rx IQ skew can be obtained with only one measurement. Correlation-based method is experimentally verified to obtain the Tx and Rx IQ skew over a large measurement range^23^. Advanced DSP algorithms on the Rx side are also applied for Tx/Rx impairment separation, such as using 4×4 and 8×2 multiple-input multiple-output (MIMO) digital adaptive equalizer (AEQ) to obtain the Tx/Rx XY skew or Tx/Rx IQ skew^24,25^. We previously proposed a scheme in^26^ that can calibrate the Tx/Rx IQ skew and Tx/Rx AFR. However, all these above-mentioned schemes are insufficient to calibrate all the Tx/Rx sides FDI simultaneously, which hinders further improvement of transmission performance.

In the following, we extend our oral work presented at the Optical Fiber Communication Conference 2023^27^, to propose and experimentally demonstrate a precise and multi-functional FDI calibration method based on the orthogonal separation and frequency domain analysis. To the best of our knowledge, it is the first scheme that can avoid the complex time-domain noise and simultaneously separate and obtain all FDI mentioned before. To achieve orthogonal separation, a novel specially designed interleaved multi-tone signal is used. After frequency domain analysis, the AFR, PFR, IQ skew, and XY skew of the Tx and Rx sides can be obtained. The simulation and experimental results verify the versatility and portability of the calibration scheme. The absolute estimation error on Tx/Rx side AFR/PFR is less than 1 dB/0.2 rad, and less than 0.2 ps for time skew. Benefiting from the proposed calibration, a broader-band 96 GBaud dual-polarization (DP) Nyquist 16QAM signal in back-to-back (B2B) transmission can be achieved by 64 GBaud-class 400 Gbps silicon photonics-based CO-TRx, and the measured bit error rate (BER) performance can be improved from 1.21e-1 to 4.41e-2 after FDI calibration.

## Results and discussion

### To evaluate the accuracy of proposed scheme

**Fig. 1:**
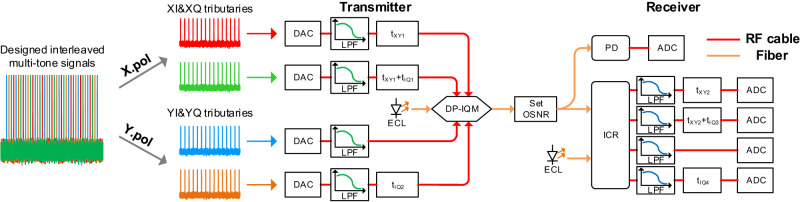
The simulation setup of the preset transmission system calibrated by using specially designed multi-tone signals. DAC digital to analog converter, LPF low-pass filter, PD photo-diode, ICR integrated coherent receiver, ADC analog to digital converter, RF radio-frequency. Red line RF cable, Original line Fiber.

Based on the VPI Transmission Maker 9.9 and MATLAB R2017a software, a typical DP CO-TRx with preset FDI is constructed as shown in Fig. 1. The link OSNR (optical signal-to-noise ratio) is set as 25 dB. The linewidth and wavelength of the optical source are set to 100 kHz and 1550 nm. AFR and PFR are simulated by using four low-pass filters, with the preset values from real optical transceivers. Noted that IQ amplitude imbalance is included in the AFR. In order to better simulate the real transmission system, Tx and Rx IQ phase imbalance are also added with values of about 5 degrees. Tx/Rx IQ skew and XY skew are generated by time-delay modules. The effective bandwidth of this CO-TRx is close to 50 GHz, and therefore the sample rate is set as 120 GSa/s.

### Calibration signal transmitted through the simulation system is analyzed

**Fig. 2:**
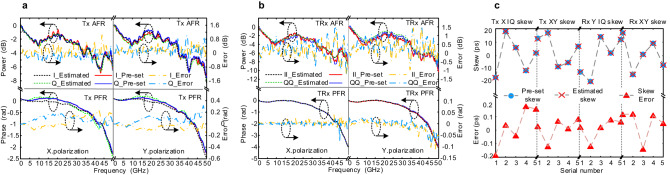
Simulation calibration results and estimated error. **a** Tx AFR and PFR in X/Y polarization states, **b** estimated TRx AFR and PFR in X/Y polarization states, **c** Tx/Rx IQ skew and XY skew in the range of −20ps ~ 20 ps. TX transmitter, TRX transceiver, RX receiver, AFR amplitude-frequency response, PFR phase-frequency response.

By using the Fourier transform process, the amplitude and phase information at the target frequency can be obtained. The simulation results include the preset and estimated AFR as well as the estimated error as shown in Fig. 2. To simplify the analysis, only the X polarization state is considered. The simulation results of Y polarization are similar and are used to calculate XY skew. At first, the Tx AFR and PFR are obtained as shown in Fig. 2a. It can be observed that the AFR estimated error is mostly less than 1 dB within 50 GHz bandwidth, and PFR estimated error is less than 0.2 rad. It should be emphasized that PFR measurement is independent of the Tx or Rx skew value, and should be nearly flat in the low-frequency domain. Similarly, the estimated errors of TRx AFR and PFR can be obtained as shown in Fig. 2b. It can be observed that the AFR absolute estimate error is less than 1 dB within 50 GHz bandwidth, except for the amplitude mutation point. The PFR absolute estimate error is less than 0.1 rad. Noted that the precision of TRx PFR is higher than Tx PFR, which is caused by the difference between the receiver modes. The Tx and Rx IQ skew are calculated from the TRx PFR in the same polarization state, and the Tx and Rx XY skew are calculated from the TRx PFR in different polarization states, as expressed in (9). Finally, the Tx/Rx IQ skew and XY skew can be obtained as shown in Fig. 2c. The preset skew values are set between −20 ps to 20 ps, and the absolute estimate error is less than 0.2 ps. Actually, the calibration range of IQ/XY skew can reach hundreds of ps.

### Calibration experiment is applied in offline CO-TRx to further verify the function and accuracy of the proposed scheme

**Fig. 3:**
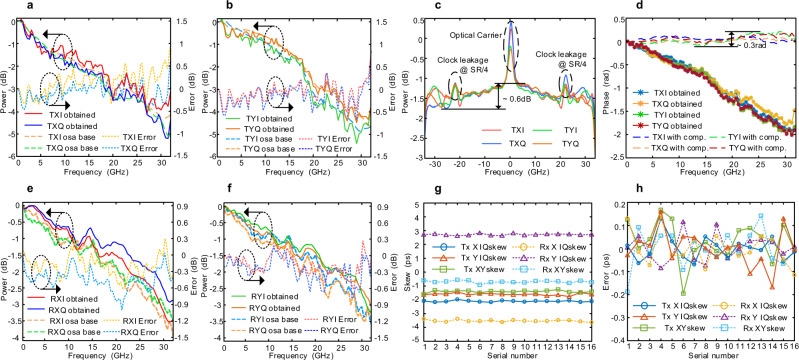
Experimental calibration results and error. **a** Obtained Tx AFR for X-polarization, **b** obtained Tx AFR for Y-polarization, **c** captured optical spectrum after Tx AFR compensation, **d** obtained Tx PFR and Tx PFR after compensation, **e** obtained Rx AFR for X-polarization, **f** obtained Rx AFR for Y-polarization, **g** obtained Tx/Rx IQ and XY skew values in 16 consecutive tests, **h** fluctuation of Tx/Rx IQ and XY skew in 16 consecutive tests. OSA optical spectrum analyzer. TX transmitter, RX receiver, AFR amplitude-frequency response, PFR phase-frequency response. SR sample rate, comp. compensation.

The experimental setup is consistent with the framework shown in Fig. 1. The link OSNR is pre-tested at about 25 dB. On the Tx side, a DP-IQ modulator (iXblue, MPZ-LN-40) with a 3 dB bandwidth of 40 GHz is used. The Tx digital to analog converters (DAC) are realized by an arbitrary waveform generator (AWG, Keysight M8196A) with a 3 dB bandwidth of 30 GHz, and the sample rate (SR) is set to 85.12 GSa/s. On the Rx side, an integrated coherent receiver (ICR, Neophotonics MicorICR Class 40) with a 3 dB bandwidth of 43 GHz is used. The Rx analog to digital converters (ADC) connected to ICR are realized by a digital oscilloscope (DSO-1, Lecroy 10-36Zi-A) with a 3 dB bandwidth of 36 GHz, the Rx ADC connected to PD is realized by a digital oscilloscope (DSO-2, Tektronics OPO73304D) with 3 dB bandwidth of 23 GHz, noted that the operation bandwidth of PD and DSO-2 are controlled within 1 GHz. The calibration results of the Tx AFR are presented in Fig. 3a, b. The base curves of Tx AFR are captured by an HR OSA from the output optical spectrum of the optical transmitter when loading a flat broadband signal.

**Fig. 4:**
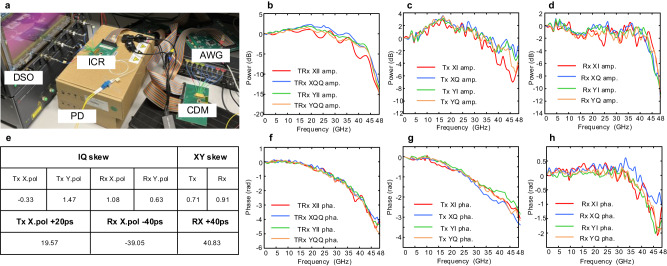
The experimental setup and calibration results. **a** Profile display of silicon photonics-based 400 Gbps CO-TRx, **b** measured TRx AFR, **c** measured Tx AFR, **d** measured Rx AFR, **e** measured original IQ/XY skew and IQ/XY skew with additional values, **f** measured TRx PFR, **g** measured Tx PFR, **h** measured Rx PFR. TRX transceiver, TX transmitter, RX receiver, AFR amplitude-frequency response, PFR phase-frequency response.

By importing a flat optical signal to the ICR, the base curves of Rx AFR can be obtained from the envelope of received signals. It can be observed that the power attenuation for Tx and Rx at 32 GHz are about 4.5 dB and 3 dB, and the absolute estimate error for the Tx/Rx side of the AFR is mostly less than 1 dB. Figure 3c gives the optical spectrum from the transmitter after compensating the Tx AFR based on the calibrated results. The optical carrier and clock leakage at the frequency of SR/4 introduced from the impairments of sub-DACs^28^ are also included. The calibration results of the Tx PFR are presented in Fig. 3d, the phase degradation at 32 GHz is about 2 rad. Since we don’t have a proper way to get the base of the PFR, the calibration accuracy is proved by the result after compensating with the calibration value, and the total fluctuation of PFR curves after compensation can be kept within 0.3 rad. The calibration results of the Rx AFR are presented in Fig. 3e, f. The base curves of Rx AFR are calculated in Rx DSP when loading a flat broadband optical signal to the ICR. The calibration of Tx/Rx IQ skew and XY skew values in 16 consecutive tests are shown in Fig. 4g. The absolute skew distribution of CO-TRx is within 4 ps, indicating that the transceiver is pre-calibrated. The fluctuation of the calibrated skew values compared to the average values is shown in Fig. 3h, and the absolute estimate error is less than 0.2 ps.

### Experimental transmission results with calibration

Figure 4a shows the experimental setup and the profile display of the 64 GBaud-class silicon photonics-based 400 Gbps CO-TRx. Two external cavity lasers with the power of 15.5 dBm and 10 dBm are used as the optical signal carrier (SC) and local oscillator (LO). The wavelength and linewidth are set as 1550 nm and 100 kHz, respectively. An AWG (Keysight M8194A) with a 3 dB bandwidth of 45 GHz is used to generate the electrical signal and to drive the coherent driver modulator. The modulated signal is then amplified by an erbium-doped fiber amplifier. At the receiver, the silicon photonics-based ICR is used to reconstruct the optical field.

The detected electrical signals are captured by a 256 GSa/s real-time oscilloscope (Keysight, UXR0704A) with operation bandwidth beyond 70 GHz.

During the FDI calibration process, the original value of frequency interval ${{{{{{\rm{f}}}}}}}_{0}$, sub-frequency interval $\varDelta f$ and N are set as 750 MHz, 1.875 MHz, and 64, respectively to cover the 50 GHz bandwidth. The Fig. 4b–d shows the measured TRx, Tx, and the calculated Rx AFR, and Fig. 4f–h shows the measured TRx, Tx, and the calculated Rx PFR, respectively. It can be observed that the frequency response for each tributary is inconsistent, and the ripples on the response curves are also obvious. Meanwhile, the curves of Rx AFR and Rx PFR are relatively flat within 35 GHz. However, the curves suddenly drop down when the frequency is higher than 35 GHz, which is the most common FDI encountered in ultra-wideband transmission scenarios. Figure 4e shows the measured original IQ/XY skew values, and the additional skew values are applied in different input/output ports to verify the calibration accuracy. The fluctuation of the measured skew is within ±0.2 ps.

### To evaluate the transmission performance improvement

**Fig. 5:**
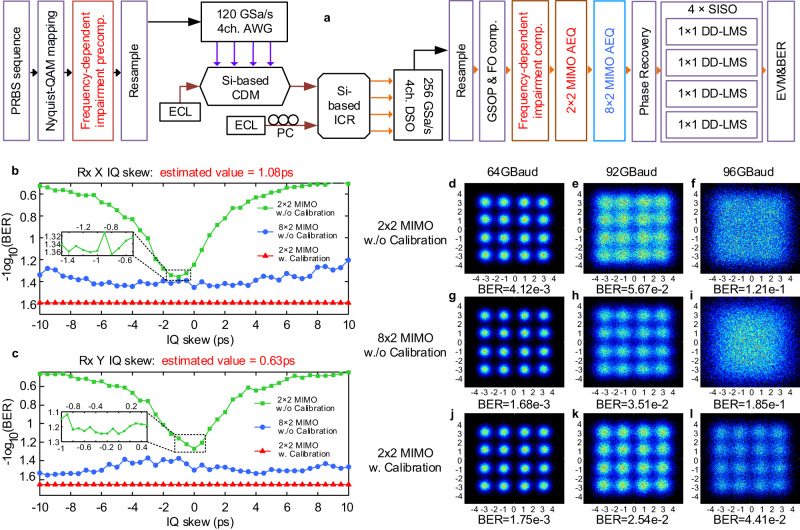
The experimental setup and transmission results. **a** The flow chart of equalization algorithms (the compensation module with calibration process is in red, the compensation module without calibration process is in blue, the rest are the same). Transmission performance of the received 92GBaud DP Nyquist 16QAM signal under different conditions when scanning **b** Rx X IQ skew (using 2×2 MIMO and without calibration process in green line, using 8×2 MIMO and without calibration process in blue line, using 2×2 MIMO and with calibration process in red line), **c** Rx Y IQ skew. Constellation diagrams for 64/92/96 GBaud DP Nyquist 16QAM signals, **d/e/f** without calibration process and using 2×2 MIMO, **g/h/i** without calibration process and using 8×2 MIMO, **j/k/l** with calibration process and using 2×2 MIMO. AWG arbitrary waveform generator, CDM coherent driver modulator, ECL external cavity laser, ICR integrated coherent receiver, DSO digital oscilloscope, GSOP Gram-Schmidt orthogonalization procedure, FO comp. frequency offset compensator, AEQ adaptive equalizer, MIMO multiple-input multiple-output, SISO single input single output, Rx receiver. EVM error vector magnitude, BER bit error rate.

The BER of 64/92/96 GBaud DP Nyquist 16QAM signals are measured in terms of different conditions. The experimental setup and the flow chart of equalizers are shown in Fig. 5a. In the Rx DSP module, whether using or not the calibration process, conventional coherent algorithms, including Gram-Schmidt orthogonalization procedure, laser frequency offset compensator, phase recovery, and four blind 1 × 1 DD-LMS single input single output equalizers are used to help demodulate the signal. For compensation flow with calibration process (in red), static FDI pre-/post- compensators are applied based on the measured AFR/PFR and IQ/XY skew values. Only a low-complexity 2 × 2 data-aided MIMO (multi-input and multi-output) adaptive equalizer is used to de-mux the polarization crosstalk induced by RSOP. Without the calibration process, we use a high-complexity 8 × 2 data-aided MIMO adaptive equalizer (in dashed blue line) for comparison. The tap length of the 2 × 2 MIMO AEQ is 49, and the tap length of the 8 × 2 MIMO AEQ is 43. Therefore, according to^24^, the computational complexity in Rx DSP is reduced by 75% with the proposed calibration method.

In Fig. 5b, c, the transmission performance of 92 GBaud DP 16QAM signals under different conditions when scanning Rx IQ skew are presented. It can be observed the BER of 2 × 2 MIMO without calibration degrades rapidly with the increase of Rx IQ skew. But the BER of 8 × 2 MIMO without calibration and 2 × 2 MIMO with calibration is not sensitive to Rx IQ skew because of their better compensation effect. Subsequently, due to the benefits from AFR/PFR compensation, the BER performance of 2 × 2 MIMO with calibration is better than 8 × 2 MIMO without calibration. Actually, the scanning process itself can approximately obtain the Rx IQ skew, which is shown in the depression of the green curve. However, when the accuracy of the scanning is increased as shown in the small box in Fig. 5b, c, the BER performance will exhibit a flat interval with a width of 0.8 ps, indicating that the error of this method is about 0.4 ps. And the estimated IQ skew values of our proposed method are exactly within this flat interval, which further proves the accuracy of the calibration process. The difference in BER between the green and blue curves with Rx IQ skew compensation is caused by the Tx IQ skew (−0.33/1.47 ps for X/Y polarization state), which can also be verified by our calibration results. The calibrated value of the Tx X IQ skew is −0.33 ps, thus the BER performance in these two conditions with Rx IQ skew compensation is very close. As the calibrated value of Tx Y IQ skew is 1.47 ps, this value will result in about 0.2 dB BER performance difference between 2×2 MIMO and 8×2 MIMO without calibration.

The constellations of the recovered DP Nyquist 16QAM signals in different situations are proposed in Fig. 5d–l, respectively. For 64 GBaud transmission, it can be observed that the BER performance of 2×2 MIMO without calibration is a little worse than the other two conditions. The reason is that the FDI impact of this 400 Gbps optical coherent transceiver is small for the 64 GBaud signal transmission. And the BER performance is very close comparing the 8×2 MIMO without calibration and 2×2 MIMO with calibration. However, for 92 GBaud transmission, the BER performance of the three conditions presents a stepped distribution, the compensation effect of 2×2 MIMO with calibration process is obviously better than that of 8×2 MIMO without calibration. Finally, for 96 GBaud transmission, the recovered constellations of 2×2 MIMO and 8×2 MIMO without calibration are both bunched up and cannot distinguish the constellation points, the BER also illustrates the failure of signal demodulation. However, the constellation points can be distinguished by using the 2×2 MIMO with calibration process, and the measured BER value of 4.41e-2 can be achieved, which is less than the 24% overhead soft-decision forward error correction (SD-FEC) threshold of 4.5e-2. These experiment results further illustrate the necessity of the calibration process for a high baud rate optical transmission system and the effectiveness of our proposed calibration method.

## Conclusions

We have proposed and experimentally demonstrated a precise and multi-functional FDI calibration method. With the capability of simultaneously separating and calibrating the FDI, including AFR, PFR, IQ skew, and XY skew from Tx and Rx sides. Under the condition of channel OSNR of about 25 dB, the absolute estimate error of the AFR and PFR for both Tx/Rx sides are less than 1 dB and 0.2 rad in the target frequency domain, respectively. The absolute estimate error of the IQ skew and XY skew for both Tx/Rx sides is less than 0.2 ps. The proposed scheme is confirmed experimentally in a silicon photonics-based 400 Gbps optical coherent transceiver by transmitting 64/92/96 GBaud DP Nyquist 16QAM signals in different conditions. The measured BER is significantly improved after calibration. Finally, 96 GBaud DP Nyquist 16QAM signals over B2B transmission are achieved with the measured BER value below the 24% SD-FEC threshold, the measured BER value can be improved from 1.21e-1 to 4.41e-2 by using the proposed FDI calibration scheme. These experimental results indicate the potential of the proposed method as a suitable calibration scheme to mitigate the inherent FDI for high-speed CO-TRx and reveal the ability being applied in CO-TRx with a capacity beyond 800 Gbps/λ. In the future, we will continue to improve this method and strive to achieve low cost at the chip level.

## Methods

### An example of the design flow of multitone signal in simulation is as follows

1. The signal length should be set to an integer multiple of 512 to meet the RAM requirement and can be set as 512×500 = 256,000.
2. With the relation of 120e9/256,000 = 468,750, we define the 468,750 Hz as base frequency ${{{{{{\rm{f}}}}}}}_{base}$, and all the frequency components in (5) should be the integer multiple of ${{{{{{\rm{f}}}}}}}_{base}$ to guarantee the integrality of each tone signal.
3. The number of tones in each tributary N is set as 64 to consider both bandwidth resolution and signal power.
4. Original value of frequency interval ${{{{{{\rm{f}}}}}}}_{0}$ is set as 1600×${{{{{{\rm{f}}}}}}}_{base}$ = 750 MHz to meet the 50 GHz bandwidth.
5. Original value of subfrequency interval $\varDelta f$ is set as 4×${{{{{{\rm{f}}}}}}}_{base}$ = 1.875 MHz.
6. With this setup, the maximum calibration frequency received by the ICR is about 52.43 GHz, and the maximum frequency received by the PD is about 870 MHz.
  In the experimental operation, we need to pay attention to the following aspects:
7. Avoiding excessive radio frequency signal power. When the modulator inputs an excessively high-power signal, it will cause significant nonlinear distortion and lead to the failure of the multitone signal-based calibration.
8. By appropriately reducing the number of tones in a multitone signal, the signal-to-noise ratio can be improved, thereby improving calibration accuracy under low OSNR conditions.
9. Due to the laser frequency offset effect, two different tones may overlap exactly within the 10 MHz frequency domain, resulting in invalid phase information obtained at this time.
10. Clock line may appear at the quarter sampling rate of the transmitter, thereby the design of the multi-tone signal should avoid generating signals near this frequency.

### Impairment model of coherent optical transceiver

The FDI in CO-TRx mainly comes from four factors:

1. Amplitude/phase response imperfections in electronic and optical components, including different gain, ripple, and phase characteristics in the variance of frequencies,
2. Effect of bias voltage deviation in modulator, hybrid, etc.,
3. Time skew between different transmission ports,
4. Joint effect of laser frequency offset, phase noise, azimuth rotation, and RSOP.

In fact, factor 4 is not the impairment of the device itself, nor can it be calibrated, but it is the main factor that causes aliasing of impairments from different ports and Tx/Rx sides. Factors 1 ~ 3 can be regarded as the inherent FDI of the CO-TRx to be calibrated, and will equally occur on both Tx and Rx sides. When modeling the FDI in CO-TRx, the relationship between the above four factors should be clearly sorted out.

In our previous work^22^, the preliminary impairment model of CO-TRx was derived in the frequency domain. In this chapter, we will supplement the results of the preliminary model and consider more FDI. The DP FDI model with the effect of Tx/Rx side XY skew is added in the appendix. To simplify the formula, a single polarization state model is considered here. The impairment model of the optical transmitter ${H}_{Tx}(\omega )$ is:

1 $${H}_{Tx}(\omega )=\left(\begin{array}{cc}{a}_{TI}(\omega ){e}^{i({\varphi }_{TI}(\omega ))} & \sin ({\beta }_{1}){a}_{TQ}(\omega ){e}^{i({\varphi }_{TQ}(\omega )-\omega {\tau }_{1})}\\ 0 & \cos ({\beta }_{1}){a}_{TQ}(\omega ){e}^{i({\varphi }_{TQ}(\omega )-\omega {\tau }_{1})}\end{array}\right).$$

Here, ${a}_{Tm}(\omega )/{\varphi }_{Tm}(\omega )$ (m = I or Q) represents the AFR/PFR of the transmitter from the I or Q tributary. ${\tau }_{1}$ and ${\beta }_{1}$ represent the values of Tx IQ skew and Tx IQ phase imbalance.

The impairment model of the optical receiver ${H}_{Rx}(\omega )$ can also be derived. As the receiver includes the beating effect of the SC and LO, ${H}_{Rx}(\omega )$ should consider the influence of laser frequency offset, phase noise, and azimuth rotation. The formula of ${H}_{Rx}(\omega )$ is given in (2). Similarly, ${a}_{Rm}(\omega )/{\varphi }_{Rm}(\omega )$ (m = I or Q) represents the AFR/PFR of the receiver from I or Q tributary. ${\tau }_{2}$ and ${\beta }_{2}$ represent the values of Rx IQ skew and Rx IQ phase imbalance. ${\varLambda }_{amp}(\omega )/{\varLambda }_{\theta }(\omega )$ is the amplitude/phase envelope of the laser phase noise in the frequency domain $\varLambda (\omega )$. The phase noise from two external cavity lasers with linewidth <100 kHz, ${\varLambda }_{amp}(\omega )$ is a narrow pulse with most of the power distributed within 10 MHz bandwidth. ${\varLambda }_{\theta }(\omega )$ is a random variable dependent on frequency. ${\omega }_{O}$ is the value of frequency offset, $\theta$ is the azimuth rotation which represents the slow drift phase difference between SC and LO.

2 $${H}_{Rx}(\omega )={\varLambda }_{amp}(\omega ){e}^{i{\varLambda }_{\theta }(\omega )}\otimes \left(\begin{array}{cc}\cos (\theta ){a}_{RI}(\omega -{\omega }_{O}){e}^{i({\varphi }_{RI}(\omega -{\omega }_{O}))}\hfill & -\,\sin (\theta ){a}_{RI}(\omega -{\omega }_{O}){e}^{i({\varphi }_{RI}(\omega -{\omega }_{O}))}\hfill\\ \sin (\theta +{\beta }_{2}){a}_{RQ}(\omega -{\omega }_{O}){e}^{i({\varphi }_{RQ}(\omega -{\omega }_{O})-(\omega -{\omega }_{O}){\tau }_{2})} & \cos (\theta +{\beta }_{2}){a}_{RQ}(\omega -{\omega }_{O}){e}^{i({\varphi }_{RQ}(\omega -{\omega }_{O})-(\omega -{\omega }_{O}){\tau }_{2})}\hfill\end{array}\right).$$

3 $$\begin{array}{c}{H}_{TRx}(\omega )={\varLambda }_{amp}(\omega ){e}^{i{\varLambda }_{\theta }(\omega )}\otimes \hfill\\ \left(\begin{array}{cc}\cos (\theta ){a}_{TI}(\omega ){a}_{RI}(\omega -{\omega }_{O}){e}^{i({\varphi }_{TI}(\omega )+{\varphi }_{RI}(\omega -{\omega }_{O}))}\hfill & -\,\sin (\theta -{\beta }_{1}){a}_{TQ}(\omega ){a}_{RI}(\omega -{\omega }_{O}){e}^{i({\varphi }_{TQ}(\omega )+{\varphi }_{RI}(\omega -{\omega }_{O})-\omega {\tau }_{1})}\hfill \\ \sin (\theta +{\beta }_{2}){a}_{TI}(\omega ){a}_{RQ}(\omega -{\omega }_{O}){e}^{i({\varphi }_{TI}(\omega )+{\varphi }_{RQ}(\omega -{\omega }_{O})-\omega {\tau }_{2}+{\omega }_{O}{\tau }_{2})} & \cos (\theta +{\beta }_{2}-{\beta }_{1}){a}_{TQ}(\omega ){a}_{RQ}(\omega -{\omega }_{O}){e}^{i({\varphi }_{TQ}(\omega )+{\varphi }_{RQ}(\omega -{\omega }_{O})-\omega ({\tau }_{1}+{\tau }_{2})+{\omega }_{O}{\tau }_{2})}\hfill\end{array}\right).\end{array}$$

By using ${H}_{TRx}(\omega )={H}_{Rx}(\omega ){H}_{Tx}(\omega )$, the FDI model of the CO-TRx in the frequency domain is presented in (3). It can be observed that the effect of IQ phase imbalance and azimuth rotation will together cause the aliasing between channels of the transmitter and receiver. Therefore, it is rather difficult to directly separate and calibrate all the FDI from ${H}_{TRx}(\omega )$.

### Operation principle of the proposed calibration method

To realize the orthogonal separation scheme based on frequency domain analysis, specially designed multi-tone signals are proposed. Noted that multi-tone signal is just one implementation to realize this scheme, but not the only solution. The multi-tone signal is chosen for its strict orthogonality in frequency domain, flexibility in signal design, and possibility of transmission with the payload signal.

The main idea of our proposal is to measure the Tx FDI by multi-tone beating with a low-bandwidth PD ( < 1 GHz) implemented at the Tx side, and to measure the overall TRx FDI by field reconstruction with the assistance of the tested CO-TRx itself. Therefore, the specially designed multi-tone signals should be able to separate the impairments whether received by using PD or CO-TRx. In our scheme, the transmitted multi-tone signals can be described as:

4 $${{{{{{\rm{X}}}}}}}_{\eta }({{{{{\rm{t}}}}}})=\mathop{\sum }\limits_{{{{{{\rm{k}}}}}}=1}^{{{{{{\rm{N}}}}}}}\left\{\cos \left[2\pi {{{{{{\rm{f}}}}}}}_{\eta }({{{{{\rm{k}}}}}}){{{{{\rm{t}}}}}}+{\varPhi }_{\eta }({{{{{\rm{k}}}}}})\right]\right\},$$

where ${{{{{{\rm{f}}}}}}}_{\eta }(k)$ with $\eta \in \{XI,XQ,YI,YQ\}$ represents the frequency of tone signals, ${\varPhi }_{\eta }(k)$ is the preset random phase used to reduce the peak-to-average power ratio of the signal^19^. N is the number of tones transmitted in each tributary and is set according to the target calibration bandwidth. More specifically, the key frequency parameter is designed as:

5 $$\left\{\begin{array}{c}\hskip -40pt{{{{{{\rm{f}}}}}}}_{{{{{{\rm{XI}}}}}}}({{{{{\rm{k}}}}}})={{{{{{\rm{kf}}}}}}}_{0}+\varOmega ({{{{{\rm{k}}}}}}),\,{{{{{{\rm{f}}}}}}}_{{{{{{\rm{XQ}}}}}}}({{{{{\rm{k}}}}}})={{{{{{\rm{f}}}}}}}_{{{{{{\rm{XI}}}}}}}({{{{{\rm{k}}}}}})+\frac{2}{4}({{{{{{\rm{f}}}}}}}_{0}+\varOmega ({{{{{\rm{k}}}}}})),\\ {{{{{{\rm{f}}}}}}}_{{{{{{\rm{YI}}}}}}}({{{{{\rm{k}}}}}})={{{{{{\rm{f}}}}}}}_{{{{{{\rm{XI}}}}}}}({{{{{\rm{k}}}}}})+\frac{1}{4}({{{{{{\rm{f}}}}}}}_{0}+\varOmega ({{{{{\rm{k}}}}}})),\,{{{{{{\rm{f}}}}}}}_{{{{{{\rm{YQ}}}}}}}({{{{{\rm{k}}}}}})={{{{{{\rm{f}}}}}}}_{{{{{{\rm{XI}}}}}}}({{{{{\rm{k}}}}}})+\frac{3}{4}({{{{{{\rm{f}}}}}}}_{0}+\varOmega ({{{{{\rm{k}}}}}})).\end{array}\right.$$

**Fig. 6:**
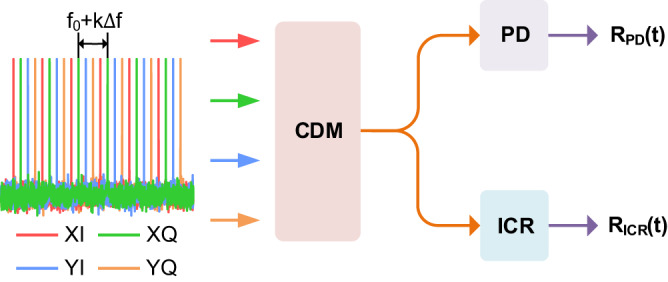
Schematic diagram of specially designed multi-tone signals. The signal after PD/ICR detection are used to obtain the impairments from Tx/TRx side. CDM coherent driver modulator, PD photodiode, ICR integrated coherent receiver, TX transmitter, TRX transceiver.

Here, the frequency interval between two adjacent tones in one tributary is ${{{{{{\rm{f}}}}}}}_{0}+k\varDelta f$, and ${{{{{{\rm{f}}}}}}}_{0}$ is the primary value. $\varOmega (k)$ is defined as the sub-frequency interval with $\varOmega (k+1)-\varOmega (k)=k\varDelta f$, where $\varDelta f$ is the original value of $\varOmega (k)$. The schematic diagram of the specially designed multi-tone signal is shown in Fig. 6. Obviously that the four calibration signals are interleaved in the frequency domain. The existence of sub-frequency interval ensures the interleaving characteristic of multi-tone signals after being detected by a single PD. Assuming that the optical IQ modulator is biased at the linear point, the signal after PD detection can be expressed as:

6 $${{{{{{\rm{R}}}}}}}_{{{{{{\rm{PD}}}}}},\eta }({{{{{\rm{f}}}}}})=\chi \mathop{\sum }\limits_{{{{{{\rm{k}}}}}}=1}^{{{{{{\rm{N}}}}}}}{{{{{{\rm{a}}}}}}}_{{{{{{\rm{Tx}}}}}},\eta }({{{{{\rm{k}}}}}})\cdot {{{{{{\rm{p}}}}}}}_{{{{{{\rm{Tx}}}}}},\eta }({{{{{\rm{k}}}}}})\cdot \delta [2\pi ({{{{{\rm{f}}}}}}-({{{{{{\rm{f}}}}}}}_{0}+{{{{{\rm{k}}}}}}\varDelta {{{{{\rm{f}}}}}}))],$$

where $\eta =XI/XQ/YI/YQ$, $\chi$ is a normalization constant, and $\delta$ is the Dirac delta function. ${a}_{Tx,\eta }(k)$ can be approximated as the amplitude response at the frequency $[{f}_{\eta }(k)+{f}_{\eta }(k+1)]/2$ and ${p}_{Tx,\eta }(k)$ can be approximated as the phase difference between the frequency ${f}_{\eta }(k)$ and ${f}_{\eta }(k+1)$. The Tx PFR ${\varphi }_{Tx,\eta }(k)$ is calculated from the integral operation of ${p}_{Tx,\eta }(k)$, which can be expressed as ${\varphi }_{Tx,\eta }(k+1)={\varphi }_{Tx,\eta }(k)+{p}_{Tx,\eta }(k)$. Noted that the group delay induced by the integration operation can be eliminated after obtaining the Tx IQ skew. The received signals obtained by the coherent optical receiver can be described as:

7 $${{{{{{\rm{R}}}}}}}_{{{{{{\rm{ICR}}}}}},\eta }({{{{{\rm{t}}}}}})= 	 \,{\alpha }_{{{{{{\rm{pol}}}}}}\cdot {{{{{\rm{X}}}}}}}\Big\{{\chi }_{{{{{{\rm{XI}}}}}},\eta }\mathop{\sum }\limits_{{{{{{\rm{k}}}}}}=1}^{{{{{{\rm{N}}}}}}}{{{{{{\rm{a}}}}}}}_{{{{{{\rm{XI}}}}}},\eta }({{{{{\rm{k}}}}}})\cos [2\pi {{{{{{\rm{f}}}}}}}_{{{{{{\rm{XI}}}}}}}({{{{{\rm{k}}}}}}){{{{{\rm{t}}}}}}+{\varPhi }_{{{{{{\rm{XI}}}}}},\eta }({{{{{\rm{k}}}}}})+{\varphi }_{{{{{{\rm{XI}}}}}},\eta }({{{{{\rm{k}}}}}})]\\ 	 +{\chi }_{{{{{{\rm{XQ}}}}}},\eta }\mathop{\sum }\limits_{{{{{{\rm{k}}}}}}=1}^{{{{{{\rm{N}}}}}}}{{{{{{\rm{a}}}}}}}_{{{{{{\rm{XQ}}}}}},\eta }({{{{{\rm{k}}}}}})\cos [2\pi {{{{{{\rm{f}}}}}}}_{{{{{{\rm{XQ}}}}}}}({{{{{\rm{k}}}}}}){{{{{\rm{t}}}}}}+{\varPhi }_{{{{{{\rm{XQ}}}}}},\eta }({{{{{\rm{k}}}}}})+{\varphi }_{{{{{{\rm{XQ}}}}}},\eta }({{{{{\rm{k}}}}}})]\Big\}\\ 	+{\alpha }_{{{{{{\rm{pol}}}}}}.{{{{{\rm{Y}}}}}}}\Big\{{\chi }_{{{{{{\rm{YI}}}}}},\eta }\mathop{\sum }\limits_{{{{{{\rm{k}}}}}}=1}^{{{{{{\rm{N}}}}}}}{{{{{{\rm{a}}}}}}}_{{{{{{\rm{YI}}}}}}\eta }({{{{{\rm{k}}}}}})\cos [2\pi {{{{{{\rm{f}}}}}}}_{{{{{{\rm{YI}}}}}}}({{{{{\rm{k}}}}}}){{{{{\rm{t}}}}}}+{\varPhi }_{{{{{{\rm{YI}}}}}},\eta }({{{{{\rm{k}}}}}})+{\varphi }_{{{{{{\rm{YI}}}}}},\eta }({{{{{\rm{k}}}}}})]\\ 	 +{\chi }_{{{{{{\rm{YQ}}}}}},\eta }\mathop{\sum }\limits_{{{{{{\rm{k}}}}}}=1}^{{{{{{\rm{N}}}}}}}{{{{{{\rm{a}}}}}}}_{{{{{{\rm{YQ}}}}}},\eta }({{{{{\rm{k}}}}}})\cos [2\pi {{{{{{\rm{f}}}}}}}_{{{{{{\rm{YQ}}}}}}}({{{{{\rm{k}}}}}}){{{{{\rm{t}}}}}}+{\varPhi }_{{{{{{\rm{YQ}}}}}},\eta }({{{{{\rm{k}}}}}})+{\varphi }_{{{{{{\rm{YQ}}}}}},\eta }({{{{{\rm{k}}}}}})]\Big\},$$

where ${\alpha }_{Pol,X/Y}$ is distributed power for different polarization states. ${\chi }_{\eta 1,\eta 2}({\eta }_{1},\,{\eta }_{2}\in \{XI,XQ,YI,YQ\})$ represents power from different transmitter and receiver tributaries, with detailed expressions in^29^. Similarly, ${a}_{\eta 1,\eta 2}$ and ${\varphi }_{\eta 1,\eta 2}$ represent the TRx AFR and PFR respectively. In the presence of frequency offset $2\pi {f}_{0}$, the Rx AFR/PFR can be derived from the expression:

8 $$\begin{array}{c}{{{{{{\rm{a}}}}}}}_{\eta 1,\eta 2}({{{{{\rm{f}}}}}})={{{{{{\rm{a}}}}}}}_{{{{{{\rm{Tx}}}}}},\eta 1}({{{{{\rm{f}}}}}})\cdot {{{{{{\rm{a}}}}}}}_{{{{{{\rm{RX}}}}}},\eta 2}({{{{{\rm{f}}}}}}-2\pi {{{{{{\rm{f}}}}}}}_{0})\\ {\varphi }_{\eta 1,\eta 2}({{{{{\rm{f}}}}}})={\varphi }_{{{{{{\rm{Tx}}}}}},\eta 1}({{{{{\rm{f}}}}}})\cdot {\varphi }_{{{{{{\rm{RX}}}}}},\eta 2}({{{{{\rm{f}}}}}}-2\pi {{{{{{\rm{f}}}}}}}_{0})\end{array}.$$

The Tx/Rx IQ skew of X/Y polarization and Tx/Rx XY skew are derived from the TRx PFR ${\varphi }_{\eta 1,\eta 2}$, with the expression as:

9 $$\left\{\begin{array}{l}{{{{{\rm{TxXIQskew}}}}}}=[{{{{{\rm{d}}}}}}({\varphi }_{{{{{{\rm{XI}}}}}},{{{{{\rm{XI}}}}}}}-{\varphi }_{{{{{{\rm{XQ}}}}}},{{{{{\rm{XI}}}}}}})+{{{{{\rm{d}}}}}}({\varphi }_{{{{{{\rm{XI}}}}}},{{{{{\rm{XQ}}}}}}}-{\varphi }_{{{{{{\rm{XQ}}}}}},{{{{{\rm{XQ}}}}}}})]/2{{{{{\rm{df}}}}}},\\ {{{{{\rm{RxXIQskew}}}}}}=[{{{{{\rm{d}}}}}}({\varphi }_{{{{{{\rm{XI}}}}}},{{{{{\rm{XI}}}}}}}-{\varphi }_{{{{{{\rm{XI}}}}}},{{{{{\rm{XQ}}}}}}})+{{{{{\rm{d}}}}}}({\varphi }_{{{{{{\rm{XQ}}}}}},{{{{{\rm{XI}}}}}}}-{\varphi }_{{{{{{\rm{XQ}}}}}},{{{{{\rm{XQ}}}}}}})]/2{{{{{\rm{df}}}}}},\\ {{{{{\rm{TxYIQskew}}}}}}=[{{{{{\rm{d}}}}}}({\varphi }_{{{{{{\rm{YI}}}}}},{{{{{\rm{YI}}}}}}}-{\varphi }_{{{{{{\rm{YQ}}}}}},{{{{{\rm{YI}}}}}}})+{{{{{\rm{d}}}}}}({\varphi }_{{{{{{\rm{YI}}}}}},{{{{{\rm{YQ}}}}}}}-{\varphi }_{{{{{{\rm{YQ}}}}}},{{{{{\rm{YQ}}}}}}})]/2{{{{{\rm{df}}}}}},\\ {{{{{\rm{RxYIQskew}}}}}}=[{{{{{\rm{d}}}}}}({\varphi }_{{{{{{\rm{YI}}}}}},{{{{{\rm{YI}}}}}}}-{\varphi }_{{{{{{\rm{YI}}}}}},{{{{{\rm{YQ}}}}}}})+{{{{{\rm{d}}}}}}({\varphi }_{{{{{{\rm{YQ}}}}}},{{{{{\rm{YI}}}}}}}-{\varphi }_{{{{{{\rm{YQ}}}}}},{{{{{\rm{YQ}}}}}}})]/2{{{{{\rm{df}}}}}},\hfill\\ {{{{{\rm{TxXYskew}}}}}}=\left[{{{{{\rm{d}}}}}}({\varphi }_{{{{{{\rm{XI}}}}}},{{{{{\rm{XI}}}}}}}-{\varphi }_{{{{{{\rm{YI}}}}}},{{{{{\rm{XI}}}}}}})+{{{{{\rm{d}}}}}}({\varphi }_{{{{{{\rm{XI}}}}}},{{{{{\rm{XQ}}}}}}}-{\varphi }_{{{{{{\rm{YI}}}}}},XQ})\right.\hfill\\ \left.\quad+{{{{{\rm{d}}}}}}({\varphi }_{{{{{{\rm{XI}}}}}},{{{{{\rm{YI}}}}}}}-{\varphi }_{{{{{{\rm{YI}}}}}},{{{{{\rm{YI}}}}}}})+{{{{{\rm{d}}}}}}({\varphi }_{{{{{{\rm{XI}}}}}},{{{{{\rm{YQ}}}}}}}-{\varphi }_{{{{{{\rm{YI}}}}}},YQ})\right]/4df,\hfill\\ {{{{{\rm{RxXYskew}}}}}}=\left[{{{{{\rm{d}}}}}}({\varphi }_{{{{{{\rm{XI}}}}}},{{{{{\rm{XI}}}}}}}-{\varphi }_{{{{{{\rm{XI}}}}}},{{{{{\rm{YI}}}}}}})+{{{{{\rm{d}}}}}}({\varphi }_{{{{{{\rm{XQ}}}}}},{{{{{\rm{XI}}}}}}}-{\varphi }_{{{{{{\rm{XQ}}}}}},{{{{{\rm{YI}}}}}}})\right.\hfill\\ \left.+{{{{{\rm{d}}}}}}({\varphi }_{{{{{{\rm{XI}}}}}},{{{{{\rm{YI}}}}}}}-{\varphi }_{{{{{{\rm{YI}}}}}},{{{{{\rm{YI}}}}}}})+{{{{{\rm{d}}}}}}({\varphi }_{{{{{{\rm{XI}}}}}},{{{{{\rm{YQ}}}}}}}-{\varphi }_{{{{{{\rm{YI}}}}}},YQ})\right]/4df.\hfill\end{array}\right.$$

The formulas about XY skew are derived from the DP FDI model.

### The DP FDI model of CO-TRx

Firstly, the original signals of each port can be given as: $T={[Tx{I}_{0}Tx{Q}_{0}Ty{I}_{0}Ty{Q}_{0}]}^{T},$ $R={[Rx{I}_{0}Rx{Q}_{0}Ry{I}_{0}Ry{Q}_{0}]}^{T}.$ When we consider the crosstalk in the circuit between different ports on both Tx and Rx sides according to the physical mechanism of crosstalk, the actual emitted and receiver signals with crosstalk in the circuit can be expressed in (10). Here we use ${\gamma }_{T{{{{{\rm{a}}}}}}b}/{\gamma }_{Rab}(a,b\in \{1,2,3,4\})$ to represent the crosstalk intensity from channel a to channel b. Noted that only crosstalk between adjacent ports is considered. Actually, crosstalk intensity $\gamma$ is not a constant value, it is a frequency-dependent variable that continuously increases with increasing frequency and becomes apparent in high-frequency transmission^30^.

Then, the IQ signals of each polarization state can be seen as whole parameters: $Tx={[TxITxQ]}^{T}$, $Ty={[TyITyQ]}^{T}$ $R{x}_{0}={[Rx{I}_{0}Rx{Q}_{0}]}^{T},$ $R{y}_{0}={[Ry{I}_{0}Ry{Q}_{0}]}^{T}$. The DP FDI transmission model can be expressed in (11). Here ${H}_{Tx,m}/{H}_{Rx,n}(m,n=X,Y)$ are used to represent the Tx/Rx impairment model as same as formula (1) for X and Y polarization. ${\tau }_{XY1}$ and ${\tau }_{XY2}$ represent the Tx and Rx side XY skew respectively. ${\chi }_{X}$ and ${\chi }_{Y}$ are the time-dependent intensity normalized variables for X and Y polarization and are used to introduce RSOP effects in the transmission channel. The received signals for each polarization state will mix the signals from different transmitted polarization states as in (12). And this represents the transceiver impairment model combined with the Tx and Rx FDI from different polarization states as given in (13). ${H}_{TRx,mn,ab}(m,n=X,Y)$ $(ab=II,QI,IQ,QQ)$ are the four elements in the matrix ${H}_{TRx,mn}$.

Finally, the DP FDI model of CO-TRx can be presented in (14). As given in formula (9), the Tx XY skew can be obtained by analyzing the phase difference between the $TxI$ and $TyI$ signals from the same received port. And the Rx XY skew can be obtained by analyzing the phase difference between the $RxI$ and $RyI$ from the same transmitted port. What’s more, according to formula (3), the IQ skew-induced phase terms will be exactly removed by the subtraction process, retaining only XY skew-induced phase terms.

10 $$\left[\begin{array}{c}TxI\\ TxQ\\ TyI\\ TyQ\end{array}\right] 	 =\left[\begin{array}{cccc}1 & {\gamma }_{T21} & 0 & 0\\ {\gamma }_{T12} & 1 & {\gamma }_{T32} & 0\\ 0 & {\gamma }_{T23} & 1 & {\gamma }_{T43}\\ 0 & 0 & {\gamma }_{T34} & 1\end{array}\right]\left[\begin{array}{c}Tx{I}_{0}\\ Tx{Q}_{0}\\ Ty{I}_{0}\\ Ty{Q}_{0}\end{array}\right],\\ \left[\begin{array}{c}RxI\\ RxQ\\ RyI\\ RyQ\end{array}\right] 	 =\left[\begin{array}{cccc}1 & {\gamma }_{R21} & 0 & 0\\ {\gamma }_{R12} & 1 & {\gamma }_{R32} & 0\\ 0 & {\gamma }_{R23} & 1 & {\gamma }_{R43}\\ 0 & 0 & {\gamma }_{R34} & 1\end{array}\right]\left[\begin{array}{c}Rx{I}_{0}\\ Rx{Q}_{0}\\ Ry{I}_{0}\\ Ry{Q}_{0}\end{array}\right]$$

11 $$\begin{array}{c}\left[\begin{array}{c}R{x}_{0}\\ R{y}_{0}\end{array}\right]=\left[\begin{array}{cc}{H}_{Rx,X}\cdot \exp (i\omega {\tau }_{XY2}) & {O}_{2\times 2}\\ {O}_{2\times 2} & {H}_{Rx,Y}\end{array}\right]\left[\begin{array}{cc}{\chi }_{X} & {\chi }_{Y}\\ -{\chi }_{Y} & {\chi }_{X}\end{array}\right]\left[\begin{array}{cc}{H}_{Tx,X}\cdot \exp (i\omega {\tau }_{XY1}) & {O}_{2\times 2}\\ {O}_{2\times 2} & {H}_{Tx,Y}\end{array}\right]\left[\begin{array}{c}Tx\\ Ty\end{array}\right]\\ \iff \left[\begin{array}{c}R{x}_{0}\\ R{y}_{0}\end{array}\right]=\left[\begin{array}{cc}{\chi }_{X}{H}_{Tx,X}{H}_{Rx,X}\cdot \exp (i\omega ({\tau }_{XY1}+{\tau }_{XY2})) & {\chi }_{Y}{H}_{Tx,Y}{H}_{Rx,X}\cdot \exp (i\omega {\tau }_{XY2})\\ -{\chi }_{Y}{H}_{Tx,X}{H}_{Rx,Y}\cdot \exp (i\omega {\tau }_{XY1}) & {\chi }_{X}{H}_{Tx,X}{H}_{Rx,X}\end{array}\right]\left[\begin{array}{c}Tx\\ Ty\end{array}\right].\end{array}$$

12 $$\begin{array}{c}R{x}_{0}=\left[{\begin{array}{c}Rx{I}_{0}\\ RxQ\end{array}}_{0}\right]={\chi }_{X}{H}_{Tx,X}{H}_{Rx,X}\cdot \exp (i\omega ({\tau }_{XY1}+{\tau }_{XY2}))\left[\begin{array}{c}TxI\\ TxQ\end{array}\right]+{\chi }_{Y}{H}_{Tx,Y}{H}_{Rx,X}\cdot \exp (i\omega {\tau }_{XY2})\left[\begin{array}{c}TyI\\ TyQ\end{array}\right],\\ R{y}_{0}=\left[\begin{array}{c}Ry{I}_{0}\\ Ry{Q}_{0}\end{array}\right]=-{\chi }_{Y}{H}_{Tx,X}{H}_{Rx,Y}\cdot \exp (i\omega {\tau }_{XY1})\left[\begin{array}{c}TxI\\ TxQ\end{array}\right]+{\chi }_{X}{H}_{Tx,X}{H}_{Rx,X}\left[\begin{array}{c}TyI\\ TyQ\end{array}\right].\end{array}$$

13 $${H}_{TRx,mn}={H}_{Tx,m}{H}_{Rx,n}=\left[\begin{array}{cc}{H}_{TRx,mn,II} & {H}_{TRx,mn,QI}\\ {H}_{TRx,mn,IQ} & {H}_{TRx,mn,QQ}\end{array}\right],(m,n=X,Y)$$

14 $$\left[{\begin{array}{c}Rx{I}_{0}\\ Rx{Q}_{0}\\ Ry{I}_{0}\\ RyQ\end{array}}_{0}\right]=\left[\begin{array}{llll}{\chi }_{X}{H}_{TRx,XX,II}{e}^{i\omega ({\tau }_{XY1}+{\tau }_{XY2})} & {\chi }_{X}{H}_{TRx,XX,QI}{e}^{i\omega ({\tau }_{XY1}+{\tau }_{XY2})} & {\chi }_{Y}{H}_{TRx,YX,II}{e}^{i\omega {\tau }_{XY2}} & {\chi }_{Y}{H}_{TRx,YX,QI}{e}^{i\omega {\tau }_{XY2}}\\ {\chi }_{X}{H}_{TRx,XX,IQ}{e}^{i\omega ({\tau }_{XY1}+{\tau }_{XY2})} & {\chi }_{X}{H}_{TRx,XX,QQ}{e}^{i\omega ({\tau }_{XY1}+{\tau }_{XY2})} & {\chi }_{Y}{H}_{TRx,YX,IQ}{e}^{i\omega {\tau }_{XY2}} & {\chi }_{Y}{H}_{TRx,YX,QQ}{e}^{i\omega {\tau }_{XY2}}\\ -{\chi }_{Y}{H}_{TRx,XY,II}{e}^{i\omega {\tau }_{XY1}} & -{\chi }_{Y}{H}_{TRx,XY,QI}{e}^{i\omega {\tau }_{XY1}} & {\chi }_{Y}{H}_{TRx,YY,II} & {\chi }_{Y}{H}_{TRx,YY,QI}\\ -{\chi }_{Y}{H}_{TRx,XY,IQ}{e}^{i\omega {\tau }_{XY1}} & -{\chi }_{Y}{H}_{TRx,XY,QQ}{e}^{i\omega {\tau }_{XY1}} & {\chi }_{Y}{H}_{TRx,YY,IQ} & {\chi }_{Y}{H}_{TRx,YY,QQ}\end{array}\right]\left[\begin{array}{c}TxI\\ TxQ\\ TyI\\ TyQ\end{array}\right].$$

### Supplementary information

Description of Additional Supplementary Files

Supplementary Data 1

Supplementary Data 2

Supplementary Data 3

Supplementary Data 4

## Data Availability

The authors declare that the data supporting the findings of this study are available within the paper and its supplementary information files. The source data for Figs. 2–5 is provided as Supplementary Data 1, Supplementary Data 2, Supplementary Data 3, and Supplementary Data 4, respectively.

## Code availability

The code for multi-tone calibration in this study is available from GitHub (https://github.com/DailqHust/NCE-submit-code).
