# Supplementary material for: Frequency-dependent impairment calibration and estimation for a 96 GBaud coherent optical transceiver
Source: Commun Eng. 2024 Jan 5;3:5. doi: 10.1038/s44172-023-00147-3 (PMC10955956; doi:10.1038/s44172-023-00147-3)
Supplement: Supplementary file 1 — Description of Additional Supplementary Files [file 44172_2023_147_MOESM1_ESM.pdf]

## Description of Additional Supplementary Files

**File name:** Supplementary Data 1
**Description:** Source data behind the graphs in Figure 2.

**File name:** Supplementary Data 2
**Description:** Source data behind the graphs in Figure 3.

**File name:** Supplementary Data 3
**Description:** Source data behind the graphs in Figure 4.

**File name:** Supplementary Data 4
**Description:** Source data behind the graphs in Figure 5.
